# Compliance with the surgical safety checklist in Switzerland: an observational multicenter study based on self-reported data

**DOI:** 10.1186/s13037-022-00327-8

**Published:** 2022-05-25

**Authors:** Annemarie Fridrich, Anita Imhof, David L. B. Schwappach

**Affiliations:** 1 Swiss Patient Safety Foundation, Asylstrasse 77, 8032 Zurich, Switzerland; 2 Institute of Social and Preventive Medicine (ISPM), University of Bern, Mittelstrasse 43, 3012 Bern, Switzerland

**Keywords:** Checklist, Compliance, Patient safety, Surgery, Measurement methods

## Abstract

**Background:**

Since publication of the surgical safety checklist by the WHO in 2009, it has been introduced in many hospitals. However, frequency and quality of surgical safety checklist use is often low probably limiting the effectiveness of the checklist in preventing patient harm. The focus of this study was to examine the current state of compliance with the surgical safety checklist in Switzerland and to evaluate how the data relates to international comparative data.

**Methods:**

Between November 2020 and March 2021 twelve hospitals with 15 sites collected for at least 200 surgical interventions each whether the three sections of the surgical safety checklist (Sign In, Team Time Out, Sign Out) have been applied. This data collection was part of a large quality improvement project focusing on measuring and improving compliance with the surgical safety checklist via peer observation and feedback. Descriptive statistics were used to analyze the data; chi-square tests were used to compare sub-samples.

**Results:**

The hospitals collected valid compliance data for 8622 surgical interventions. Mean compliance rate was 91% when distinguishing between the two categories applied (including partially applied) and not applied. In line with previous research, Sign In (93%) and Team Time Out (94%) sections have been applied more frequently than Sign Out (86%). All three surgical safety checklist sections have been applied in 79% of the surgical interventions, no sections in 1%.

**Conclusions:**

The results of this study indicate that the overall application of the surgical safety checklist in Switzerland can be considered high, although the completeness, especially of the Sign Out section, could be improved. At present, it seems difficult to compare compliance rates from different studies as measurement methods and definitions of compliance vary widely. A systematization and homogenization of the methodology within, but also beyond, national borders is desirable for the future.

## Background

In 2012 the Swiss Patient Safety Foundation (SPS) published as part of the national pilot program *progress! Safe surgery* a surgical safety checklist (SSC) for Switzerland in the three national languages French, German, and Italian [1, 2]. This checklist was based on the SSC of the World Health Organization (WHO) [3], but adapted for the Swiss healthcare context. Both checklists, from WHO and SPS, consist of three sections that should be applied at three critical points: The Sign In (SI) must be performed prior to induction of anesthesia, the Team Time Out (TTO) prior to skin incision, and the Sign Out (SO) prior to the patient or surgeon leaving the operating room (OR). Since publication, the SSC has been introduced in many hospitals in Switzerland, although SSC application is not mandatory. However, frequency and quality of SSC use in Switzerland is often low [2, 4–6], probably limiting the effectiveness of the SSC in preventing patient harm as several systematic reviews have shown [7–9].

A systematic review published in 2012 showed a range of compliance rates from 12 to 100% [7]. An international observational study combined with a systematic review and meta-analysis published in 2018 showed that overall SSC compliance rates vary internationally between 62.5% and 98.7% [9]. However, the compliance rates cannot be simply compared, as there are major differences in defining, measuring and interpreting compliance rates [10].

First of all, compliance can be defined in very different ways: Full compliance with the SSC may exist if the checklist has been signed by the responsible person [11], if all checklist sections are available and have been applied [12], if all of the items have been read aloud [13], etc. Second, compliance can be measured in many different ways: by internal hospital staff [14, 15] or external persons/audits [16], by analyzing data (in the case of electronic checklists [17]) or documents (in the case of paper checklists or measurement sheets) or by direct observation [12], etc. And third, most of the studies and reviews have a specific focus and limit the report of the compliance rates within specific target groups: adult [9] or pediatric surgery [18]), types of interventions (e.g., elective [6, 9, 18]), within single institutions, or single checklist sections (e.g., TTO [16, 18]) or TTO and SO [5, 6], etc.

In Switzerland, there is no regular, systematic collection of data on compliance with the SSC. Thus, in 2019 the national pilot program *progress! COM-Check*—*Safe surgery* aiming to implement a method for continuous monitoring of checklist compliance and to increase compliance with the SSC through peer audit and feedback was launched. First, documented compliance was collected by the participating hospitals themselves. Second, the hospital teams observed their colleagues applying the checklist and provided brief feedback immediately after the observation. The focus of this paper is on the documented compliance data to examine the current state of compliance with the SSC in all three language regions of Switzerland and to evaluate how this (self-collected, documentation-based) compliance data relates to international comparative data.

## Methods

### The national pilot program progress! COM-Check—Safe surgery

In 2019, the national pilot program *progress! COM-Check*—*Safe surgery* was launched in Switzerland. The main goals of the program were to implement a method for continuous monitoring of SSC compliance and to increase compliance with the SSC through peer audit and feedback. Participation in the program was voluntary. Requirement for participation was the willingness to form an interprofessional team consisting of at least three persons in leading positions in surgery, anesthesia, and nursing. The teams should assess for at least 200 surgical interventions whether the SSC has been applied and perform at least 30 in-house observations with immediate feedback on SSC application.

The program started with a kick-off event in January 2020. Between August and November 2020 each hospital team participated in a one-day training during which they practiced using the electronic data collection tools and giving feedback. Data collection took place between November 2020 and March 2021. In June 2021 first results of the data analyses were presented to and discussed with the hospital teams. The project ended in September 2021 with publication of the program material.

### Participating hospitals and their SSCs

At the start of the national pilot program *progress! COM-Check*—*Safe surgery*, 17 hospitals with 20 sites signed up for program. An initial analysis of their 24 SSCs revealed that there was a large variation between the checklists [19].

By the start of data collection, five hospitals had withdrawn from the program for various reasons, mainly pandemic-related, resulting in a final sample of twelve hospitals with 15 sites. The hospitals were very heterogeneous; for example, in addition to acute care hospitals, a children's hospital, an eye clinic and an ambulatory center participated in the program. While there were very similar in terms of general structure (e.g., all SSCs included at least the three sections SI, TTO, SO) and standard items such as *identity check*, there were considerable differences in the number of items. For the SI, the number of items ranged from 6 to 22 (median = 10), for the TTO from 9 to 22 (median = 12), and for the SO from 3 to 7 (median = 5). The shortest checklist comprised in total 18 items, while the longest checklist had 50 items.

### Data collection and monitoring

There were three options for data collection: Hospitals already recording their checklist use electronically could submit the exported data. Hospitals using paper SSCs had to compare them with the operating room (OR) program and record for each surgical intervention in an electronic tool whether the three SSC sections (SI, TTO, SO) had been applied. The third option concerned hospitals using the SSC as a visual aid (e.g., a laminated form of the SSC). These hospitals had to add paper sheets to the patient's records for the time of data collection and then enter the data into the electronic tool. For each surgical intervention, it was mandatory to record whether each of the three SSC sections had been applied. A checklist section was considered as *applied* if at least one item had been completed. Originally it was planned to distinguish only between the two categories *checklist section applied* versus *checklist section not applied*. Some hospitals asked for adding a third category *checklist section partially applied*. This third option was optional and not defined by the original program.

The electronic tool further contained some general indicators that had to be collected from all hospitals: date, time (night: after 4.59 PM or before 7.30 AM [20]), surgical discipline, type of anesthesia, planning of surgical intervention (elective/emergent), supplemented by a field for free comments. Before starting the data collection, the tool was adapted to the needs of the hospitals (e.g., selection of surgical disciplines). Some hospitals wanted to distinguish between different sites, others wanted to additionally record whether surgical procedures were performed on inpatients or outpatients, and still others whether procedures were performed by in-house surgeons or affiliated physicians. The electronic data collection tool was provided to the hospitals. It was cloud-based, and the data was additionally backed up weekly on local servers. The hospitals designated one person from their project team who had access to immediate evaluations in the electronic tool at any time.

## Data analysis

Descriptive statistics were used to analyze the data; chi-square tests were used to compare sub-samples (significance level *p* < 0.05). Cramer's V was calculated as effect size measurement differentiating between small (*V* = 0.10), medium (*V* = 0.30), and large (*V* = 0.50) effects [21].

## Results

### Sample

During the data collection period the hospitals assessed whether SSC had been applied for 8753 surgical interventions. Of these data, 131 had to be excluded due to multiple entries resulting in 8622 valid cases. This means that an average of 719 valid cases were collected per hospital. The sample was very heterogeneous in terms of language regions, disciplines, populations, day of the week, time of day, urgency of the procedure, and type of anesthesia (see Table 1).

**Table 1:** Characteristics of the sample (*N* = 8622)

Characteristics	Number of entries (%)
Type of data collection	
Paper based	6312 (73.21%)
Electronic	2310 (26.79%)
Checklist language	
German	4989 (57.86%)
French	1237 (14.35%)
Italian	2396 (27.79%)
Day of the week	
Monday-Friday	8075 (93.66%)
Saturday/Sunday	547 (6.34%)
Time of day	*(missing data: n* = *24)*
Day	7410 (85.94%)
Night^1^	1188 (13.78%)
Urgency of the procedure	
Elective	6958 (80.70%)
Urgent	1664 (19.30%)
Population^2^	
Adults	7793 (90.39%)
Children	829 (9.61%)
Surgical discipline	*(missing data: n* = *10)*
General/visceral	2177 (25.25%)
Orthopedics/traumatology	2826 (32.78%)
Gynecology/obstetrics	1210 (14.03%)
Urology	682 (7.91%)
Neurology	368 (4.27%)
Ophthalmology	350 (4.06)
Other	999 (11.59%)
Type of anesthesia	*(missing data: n* = *16)*
General	6204 (71.96%)
Regional	1756 (20.37%)
Local	492 (5.71%)
Other	154 (1.79%)

^1^after 4.59 PM or before 7.30 AM; ^2^Three hospitals collected exclusively or additionally data from pediatric surgical interventions

### Compliance data

**Fig. 1:**
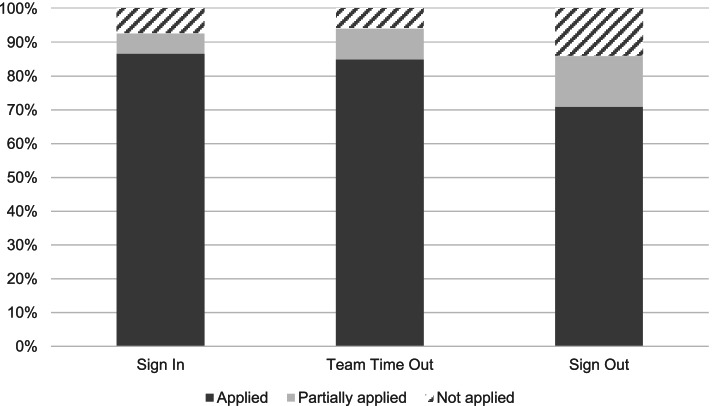
Checklist compliance for each checklist section (*N* = 8622)

The data shows that the SI was applied in 93% of all cases, the TTO in 94%, and the SO in 86% (on average 91%) when distinguishing between the two categories *applied* (including *partially applied*) and *not applied*. Of the 12 hospitals, 10 hospitals included the category *partially applied* (covering *n* = 6312 cases, 73%), while two hospitals distinguished only between *applied* and *not applied* (*n* = 2310, 27%). Considering the category *partially applied* as a separate category, data shows that the checklist sections were applied *completely* in 87% (SI), 85% (TTO), and 71% (SO) of all cases (on average 81%), and *partially* applied in 6% (SI), 9% (TTO), and 15% (SO) of all cases (see Fig. 1). For the further analyses we distinguish only between the two categories *applied* (including *partially applied*) and *not applied.*

For the SI, we found a significant difference in checklist application between elective (94%) and emergent (88%) surgical interventions (*χ*^2^(1)= 68.90, *p* <  = 0.001); effect size was medium (*V* = 0.089). This difference was also evident for the TTO (elective: 95%; emergent: 92%; *χ*^2^(1)= 19.44, *p* <  = 0.001), but here effect size was weak (*V* = 0.048). Regarding the time of the surgical interventions, there was a significant difference at the SI between day (93%) and night (90%) (*χ*^2^(1) = 23.18, *p* <  = 0.001); effect size was moderate (*V* = 0.052).

Table 2 shows all combinations of applied SSC sections.

**Table 2:** Application of checklist sections

Application of checklist sections (partly = applied)	Number (%)
No section	110 (1.28%)
Only SI	198 (2.30%)
Only TTO	94 (1.09%)
Only SO	36 (0.42%)
SI and TTO	811 (9.41%)
SI and SO	168 (1.95%)
TTO and SO	395 (4.58%)
All sections (SI, TTO, SO)	6,810 (78.98%)
Total	8622 (100%)

*SI*  Sign In, *TTO *Team Time Out, *SO *Sign Out

All three SSC sections have been applied in 79%, some sections in 20%, and no section in 1% of all cases. Comparing the samples of the surgical interventions where all three SSC sections have been applied (*n* = 6810), some checklist sections (*n* = 1702), and no section (*n* = 110), we found significant differences for urgency, type of anesthesia, day, time, and surgical discipline (see Table 3). The effects are small to medium; except for type of anesthesia where a large effect was found.

**Table 3:** Comparison of samples: three checklist sections vs. some sections vs. no section applied

Characteristic	All checklist sections applied (*n* = 6810)	Some checklist sections applied (*n* = 1702)	No checklist section applied (*n* = 110)
Urgency			
Elective	82%	78%	55%
Emergency	18%	22%	45%
* χ*^2^(2) = 60.39, *p* < = 0.001, *V* = 0.084			
Type of anesthesia			
General	75%	62%	59%
Regional	20%	22%	15%
Local	4%	10%	17%
Other	1%	6%	9%
*χ*^2^(6) = 423.89, *p* < = 0.001, *V* = 0.157			
Day			
Monday-Friday	94%	94%	85%
Saturday/Sunday	6%	7%	15%
*χ*^2^(2) = 13.03, *p* = 0.001, *V* = 0.039			
Time			
Day	86%	86%	73%
Night	14%	14%	27%
* χ*^2^(2) = 12.35, *p* = 0.002, *V* = 0.038			
Surgical discipline			
General/visceral	26%	20%	29%
Gynecology/obstetrics	14%	12%	22%
Orthopedics/traumatology	33%	31%	26%
Other	26%	37%	23%
*χ*^2^(6) = 93.80, *p* < = 0.001, *V* = 0.074			

## Discussion

The aim of this study was to assess the current state of compliance with the SSC in Switzerland via self-collected, documentation-based data and compare these data with compliance rates from other countries. With an overall compliance rate of 91%, it is considerably higher than the mean compliance rate (75%) reported in the systematic review of Borchard and colleagues [7], but in the second lowest quartile if put in the ranking of the results of Abbott and colleagues [9].

The data collection was part of a larger quality improvement initiative focusing on improving compliance with the SSC. Improvement methods were implemented at the same time as the compliance data collection, which might have had a (positive) influence on compliance data. Consistent with other findings in the literature [5, 22], the SO is the section that is least likely to be applied completely, namely in only 71% of the cases. If the SSC sections are viewed in isolation, it could be concluded that compliance at SI and TTO is already quite satisfactory, but should be improved at SO. However, a look at the entire SSC reveals that in 21% of the cases none (1%) or at least not all three checklist sections (20%) were applied. This means that in about every 5th case the compliance goal *complete SSC application of all three sections in all surgical interventions* is not met. A deeper look into the data also shows that there is a significant number of partially applied checklist sections (e.g., 9% at TTO). If we exclude this *partially applied* option, the average of the completely applied SSC sections decreases to 81% (compared to 91% including *partially applied*).

Cases with no checklist section completed at all were more likely to be emergency surgeries, especially gynecologic/obstetric procedures. This is consistent with findings from other studies [23]. A shortened checklist with a minimal set of few elementary items could be an option for such surgeries under high time pressure.

The SSCs examined in this study were very heterogeneous, as shown by a preliminary analysis at the beginning of the program [19]. Especially short SSCs are easy to use and easier to complete than longer SSCs but may not have the same effectiveness as the recommended WHO checklist. Thus, short SSCs may show better compliance rates with limited effectiveness. When measuring and comparing compliance rates, it is important to consider and report on the level of complexity of the checklists. Caution should be taken when comparing compliance data based on samples with different inclusion criteria. For example, our study included pediatric and adult, elective and emergency, weekend and weekday surgeries from different disciplines and documents varying levels of compliance within these sub-sets. Results should thus not be compared to compliance rates obtained in general elective adult surgeries which are commonly higher [24].

The measurement method seems to be a critical aspect for the comparison and interpretation of compliance rates as there are huge differences between studies [8, 10]. Thus, it is crucial to define exactly what *SSC applied* means, and to include a well-defined *SSC partially applied* category as this helps to gain a better insight of the application of the SSC sections and single items. As already requested by others [8], a uniform measurement of compliance would be desirable for the future. We thus propose the following three categories for the evaluation of paper checklists:

SSC completely applied: All SSC items have been marked as checked.

SSC partially applied: At least one SSC item has been marked as checked, but not all.

SSC not applied: No SSC item has been marked as checked or SSC is missing.

Such a definition would be most valuable for studies covering multiple sites each with different checklists, items and application procedures, such as ours.

### Limitations

The data were collected by the hospitals themselves and not validated by external controls. Therefore, we cannot make any statements about the reliability of the data. For example, we cannot verify whether all the surgical interventions for which no SSC section was applied were recorded.

The category *partially applied* was not predefined. Responses in the free comment fields indicate that individual hospitals assigned cases to categories differently. For example, some hospitals rated a missing signature as partially applied, while other hospitals rated only the application of the content items to assess the degree of fulfillment. Therefore, the data in the *partially applied* category should be interpreted with caution.

## Conclusions

The results of this study indicate that the overall application of the surgical safety checklist in Switzerland can be considered high, although the completeness, especially of the SO section, could be improved.

At the present time, there seem to be as many methods of collection as there are compliance rates. A systematization and homogenization of the methodology within, but also beyond, national borders is desirable for the future.

Finally, compliance rates tell very little about the quality of the checklists and their application [17], resulting in the risk that a pure consideration of (high) compliance rates creates a false sense of safety.

## Data Availability

The datasets used and analysed during the current study are available from the corresponding author on reasonable request.

## Abbreviations

OR: Operating Room
SI: Sign In
SO: Sign Out
SPS: Swiss Patient Safety Foundation
SSC: Surgical Safety Checklist
TTO: Team Time Out
WHO: World Health Organization
